# Multidimensional Poverty and Implementation Reach of Household Contact Tracing for Tuberculosis in South Africa: Analysis of a Randomized Trial

**DOI:** 10.1093/cid/ciag321

**Published:** 2026-06-17

**Authors:** Giridhar Mohan, Colleen F Hanrahan, Pablo Martinez-Amezcua, Christopher J Hoffmann, Patrick Biché, Neil A Martinson, David W Dowdy

**Affiliations:** Department of Epidemiology, Johns Hopkins Bloomberg School of Public Health, Baltimore, Maryland, USA; Department of Epidemiology, Johns Hopkins Bloomberg School of Public Health, Baltimore, Maryland, USA; Department of Epidemiology, Johns Hopkins Bloomberg School of Public Health, Baltimore, Maryland, USA; Department of Medicine, Johns Hopkins University School of Medicine, Baltimore, Maryland, USA; Department of Epidemiology, Johns Hopkins Bloomberg School of Public Health, Baltimore, Maryland, USA; Perinatal HIV Research Unit, Chris Hani Baragwanath Academic Hospital, Soweto, South Africa; Department of Epidemiology, Johns Hopkins Bloomberg School of Public Health, Baltimore, Maryland, USA

**Keywords:** tuberculosis, poverty, implementation science, contact investigation

## Abstract

**Background:**

Household contact tracing (HHCT) is a widely recommended intervention for tuberculosis (TB) but is poorly implemented. Multidimensional poverty, encompassing deprivations in health, education, and living standards, may hinder implementation success.

**Methods:**

Using a cluster-randomized trial in South Africa, we examined the relationship between household multidimensional poverty as a composite deprivation score and HHCT reach as (1) initiation (at least one household contact screened); (2) continuous proportion of eligible household contacts screened; and (3) completion (all eligible household contacts screened once initiated). We performed multilevel mixed-effects modeling with modified Poisson and ordered beta regression.

**Results:**

Despite near-universal comfort with HHCT (97%) among 3392 households, only 43% initiated HHCT and 19% completed screening. Greater multidimensional poverty was significantly associated with lower HHCT initiation in urban households (adjusted prevalence ratio [aPR]: 0.903, 95% confidence interval [CI]: 0.831, 0.984, per 10% higher deprivation; *P* = .021), but not in rural households (aPR: 1.06, 95% CI: 0.99, 1.13; *P* = .083). Across all households, we observed a modest but statistically significant association between greater poverty and lower predicted proportion of household contacts screened (adjusted average marginal effect [aAME]: −1.22% [−2.20%, −0.236%], per 10% higher deprivation; *P* = .015). Among households that initiated HHCT, multidimensional poverty was not significantly associated with screening completion (aPR: 0.945, 95% CI: 0.865, 1.03; *P* = .207).

**Conclusions:**

Stated comfort with HHCT may not directly translate into implementation success. Multidimensional poverty was a significant structural barrier for HHCT initiation in an urban, South African township. Once initiated, getting all household contacts screened may require targeting additional operational and health system barriers.

## Teaser

Despite near-universal comfort with household contact tracing for tuberculosis in our study of over 3000 South African households, only 1 in every 5 households screened all eligible contacts. Multidimensional poverty was a significant structural barrier to initiation in urban households.

Tuberculosis (TB) remains a major global public health challenge, responsible for an estimated 10.7 million incident persons with TB and 1.23 million deaths in 2024 [1]. One widely recommended strategy to fight the TB pandemic is household contact tracing (HHCT), which often involves a clinic-based outreach team who visit the households of individuals diagnosed with TB to screen, diagnose, and treat any contact persons who may themselves have TB [2]. Provision of TB preventive treatment (TPT) to 90% of household contact persons is 1 of 7 targets for TB control set by the 2023 United Nations high-level meeting on TB; as of 2024, only 25% of such individuals had received TPT [1]. Successful screening of household contact persons (ie, HHCT) is a necessary precondition for achieving this target. In high-TB-burden countries like South Africa, where TB incidence is estimated at 389 per 100 000 (2024) [1], there remains a disconnect between universal recommendations for HHCT and observed low levels of screening success [2–4], making it a key priority to study implementation barriers.

Evidence from South Africa has demonstrated both promise and limitations of HHCT. The large-scale ZAMSTAR cluster-randomized trial of 24 communities in Zambia and South Africa found a non-significant 18% reduction in TB prevalence over 4 years from HHCT [5]. Subsequent trials in South Africa examining different HHCT strategies, including intensified [6], incentive-based [7], and alternative-timed HHCT [8], have produced null findings with suboptimal implementation reach across urban and rural settings. This raises the question of who is being missed and why, underscoring the need to identify structural characteristics that may predict low HHCT reach in order to design targeted approaches intended to translate HHCT's epidemiologic potential into real-world impact.

Previous studies have found indicators of poverty, including lower socioeconomic status and lower educational attainment, to be associated with delayed healthcare-seeking behavior for TB [9–17]. However, whether household poverty impedes active, outreach-based HHCT, where the burden of engagement shifts from the individuals to the health system, remains understudied. The global Multidimensional Poverty Index (MPI), developed in 2010 by the University of Oxford's Poverty and Human Development Initiative and the United Nations Development Programme, measures household multidimensional poverty (MP) as acute deprivations across 3 dimensions—health, education, and living standards—using 10 indicators (Figure 1) [18]. The MPI provides a more comprehensive assessment of poverty than household income or assets scales alone, capturing additional dimensions that are likely to influence health-related behaviors (Supplementary Methods 1) [18, 19]. No previous study, to our knowledge, has examined household-level MP using the MPI to guide targeted HHCT and close the implementation gap. Therefore, we aimed to examine the relationship between household MP and reach of HHCT in the predominantly urban township of Soshanguve in the Gauteng Province and the rural districts of Vhembe and Capricorn in the Limpopo Province (Figure 2). We additionally aimed to test whether this relationship varied between urban and rural locations, considering the contextual heterogeneity, differential needs of patient populations, and health system barriers that exist.

**Figure 1.**
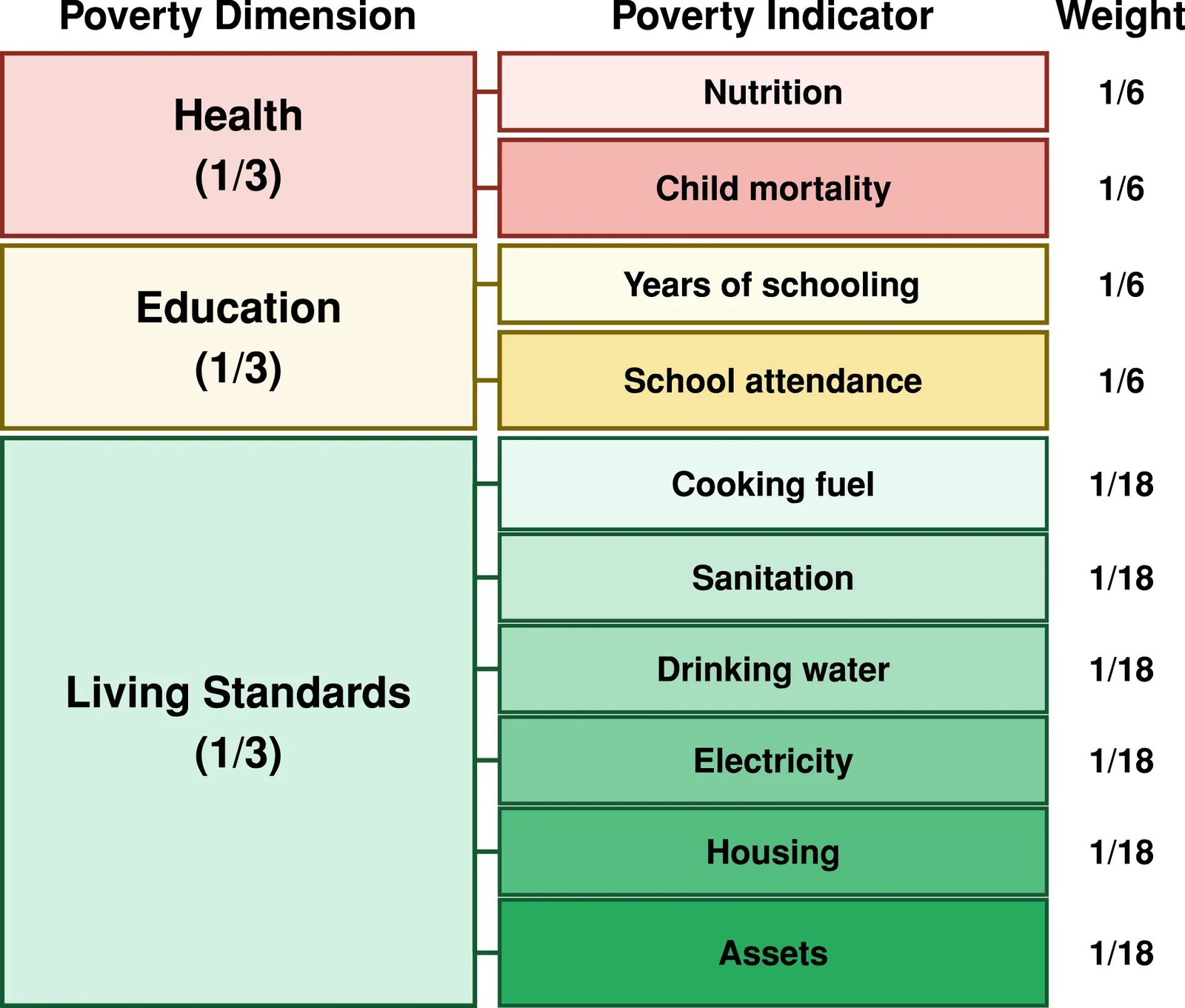
Global Multidimensional Poverty Index. The global Multidimensional Poverty Index (MPI) is an international measure of acute multidimensional poverty that has been used in over 100 low- and middle-income countries, encompassing acute deprivations across 3 dimensions using the following 10 indicators: nutrition and child mortality (Dimension #1: Health); years of schooling and school attendance (Dimension #2: Education); and cooking fuel, sanitation, drinking water, electricity, housing, and assets (Dimension #3: Standards of living) [18]. In developing an MPI score in this study, we weighted each domain as illustrated, with the exception of nutritional status, which was not measured in the study. (Child mortality—which was rare in this study [2% of households]—therefore had a weight of 1/3.)

**Figure 2.**
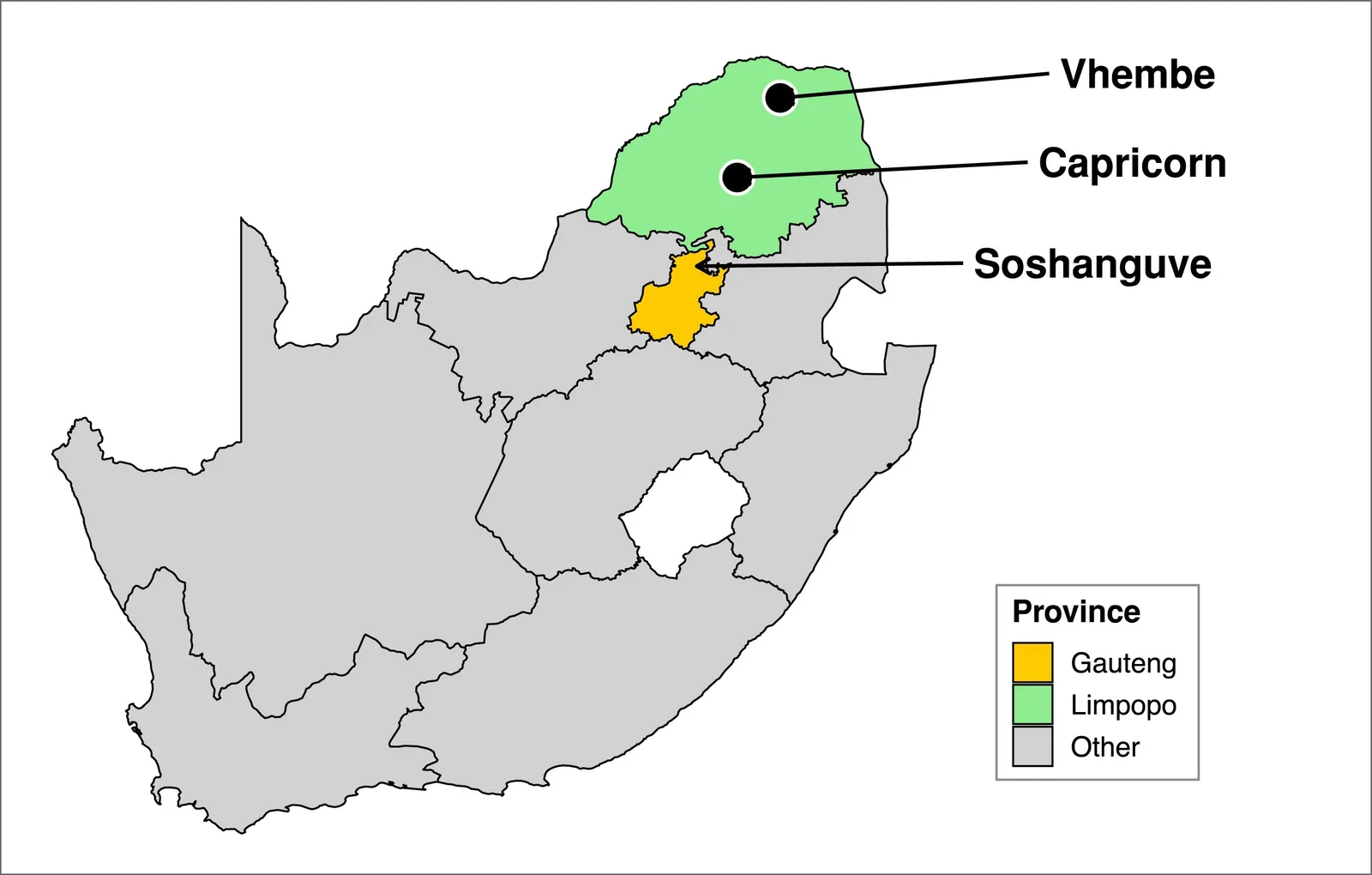
Kharituwe cluster-randomized trial sites. The Kharituwe trial in northern South Africa focused on 4417 households with individuals diagnosed with pulmonary tuberculosis from 33 primary healthcare clinics and 2 district hospitals in the predominantly urban township of Soshanguve (Gauteng Province) and 12 hospitals in the rural districts of Vhembe and Capricorn in the Limpopo Province [8].

## METHODS

### Study Design and Setting

We performed cross-sectional analysis of data from Kharituwe (“Let's go” in isiVenda), a cluster-randomized trial in South Africa examining the comparative effectiveness of 2 novel TB HHCT strategies (off-peak evening tracing in the urban Soshanguve and holiday tracing in rural Limpopo) compared to standard HHCT during weekday business hours from 8 Am to 4 Pm [8]. The investigators enrolled 4417 index participants, consisting of individuals diagnosed with pulmonary TB from 12 hospitals in Limpopo and 2 district hospitals and 33 primary healthcare clinics in Soshanguve, along with their 6150 household contacts [8]. The index participants were identified by weekly review of presumptive TB and TB treatment registers in each of these locations [8]. Household contacts consisted of individuals of all ages currently residing with or visiting the eligible index participant with TB [8].

Upon enrollment, trained study staff performed a detailed interview of all the index participants (∼30 minutes per participant) that included demographic, socioeconomic, and clinical characteristics, mobility and migration, preceding diagnostic cascade, symptom history, and assessment of costs including catastrophic health expenditures [8]. These research staff were trained to conduct the survey in the participant's choice of English or the local language in each district, paying close attention to discussing HHCT and all components of the instruments in a uniform manner across all participants [8]. Participant answers were entered directly into a mobile tablet by staff and securely transmitted into a database, which was routinely checked by the study manager for completeness [8]. At the conclusion of the interview, study staff scheduled an appointment time to arrange a household visit for consenting index participants [8].

A team of 2 individuals, including a nurse and a research assistant trained in HIV counseling and testing, performed the household visit of consenting index participants [8]. During the visit, the team members recruited household members; performed interviews on demographic, socioeconomic, mobility/migration, health-seeking behavior, and clinical characteristics; and collected sputum samples, which were sent to the National Institute for Communicable Diseases of South Africa (NICD) laboratory for Xpert Ultra TB testing [8]. The study team monitored the results of the Xpert Ultra testing using NICD's electronic laboratory system and notified household contact participants who tested positive to refer them to the nearest public-sector clinic for TB treatment [8]. Each participating household was offered a single cash payment of R150 (~$9 USD) in alignment with the South African Health Products Regulatory Authority guidelines for clinical trial participant compensation [20]. The overall study period for the parent trial was from September 2020 to August 2023 [8]. The data relevant to our analysis was collected over a period of 12 months from January 2022 to December 2022.

### Study Participants

From the original 4417 index participants, we included 3392 adult index participants (18+ years) who were diagnosed with TB in one of 42 participating health facilities and reported at least one household contact person (Supplementary Figure 1). All adult participants in the trial provided written informed consent, except for the recently deceased or critically ill, where consent was obtained from their next of kin. As the primary aim of our analysis was to examine the overall association between household MP and HHCT implementation reach, we examined all participants together to maximize statistical power and adjusted for study arm in our models to account for differences in HHCT strategy that may independently influence reach. We evaluated effect measure modification by HHCT strategy to confirm our analytic approach. The trial was registered with clinicaltrials.gov (NCT04520113) and received approval from the Institutional Review Board at Johns Hopkins Bloomberg School of Public Health and the Research Ethics Committee at the University of the Witwatersrand [8]. A Data Safety Monitoring Board was convened for the study prior to its start and then annually to provide guidance on protocol and review data for safety, progress, and efficacy [8].

### Outcome

We defined our outcome, implementation reach (success), as the proportion of named eligible household contacts in each household who were successfully screened for TB through HHCT [21, 22]. We visualized the distribution of reach, which exhibited inflated levels of zeros and ones, representing households with no reach and complete reach (ie, all named eligible household contacts screened), respectively (Supplementary Figure 2). As our objective was to comprehensively understand implementation reach across the continuum from entry into the household to completion of all eligible contacts screened, we structured our outcome as follows for analysis: (1) dichotomously as at least one household contact screened versus none to capture HHCT initiation among all households; (2) continuously as the proportion of household contacts screened to capture the extent of HHCT reach; and (3) dichotomously as achieving complete reach of all eligible household contacts screened versus not in households that initiated HHCT with some non-zero reach. We also estimated the prevalence of the comfort dimension of acceptability for HHCT as a dichotomous response to the question, “Are you comfortable with your household members being visited by our staff and tested for TB?”

### Exposure

We constructed our exposure, household MP, as a composite deprivation score based on the global MPI, which is scaled from 0 to 1, with higher scores indicating greater MP (Supplementary Methods 2). We identified measures of deprivation from detailed interviews of index participants and categorized them into 3 dimensions of poverty (health, education, living standards) with 10 corresponding indicators (Figure 1) [18]. We assigned a deprivation score of 1 if the condition was present and 0 for no deprivation. We provided equal weight of 0.33 for each of the 3 dimensions, with weighting of indicators adjusted proportionally (Supplementary Table 1). We examined missingness of data (Supplementary Tables 2–6 and Figure 3) and performed multiple imputation with chained equations across 50 imputed datasets pooled using Rubin's rule (Supplementary Methods 2 and 3) [23]. We centered MP score at its median, owing to observed right-skewness and re-scaled this variable to represent the percentage of additional indicators of deprivation (Supplementary Figure 4).

### Statistical Analysis

We used mixed-effects modified Poisson and ordered beta regression models to examine the relationship between household MP deprivation and HHCT implementation reach, while accounting for clinic-level clustering. We parameterized our mixed models with fixed effects to capture the average association across all clinics (clusters) and random effects to capture how this association varies from clinic to clinic (Supplementary Methods 4). We fit our models using maximum likelihood estimation with Laplace approximation for random effects. For HHCT initiation and completion, we used modified Poisson regression models with robust variance estimation on our dichotomous outcome to estimate prevalence ratios for initiation (at least one household contact screened vs none) and completion (all eligible household contacts screened vs not among HHCT initiated households) for each 0.1 unit (10%) higher MP deprivation from its median. For HHCT reach as a continuous proportion bounded between and including zero and one with observed inflation at both boundaries, we used an ordered beta regression, which substantially reduces over-parameterization by modeling the probability of zero and one degenerate responses alongside continuous proportions using a single linear predictor and ordered cutpoints [24]. Ordered beta regression has greater efficiency in handling zero and one inflation with higher precision in marginal effect estimates [24]. We estimated the marginal effect of MP deprivation on the predicted proportion of eligible contacts screened using numerical differentiation for each household and then averaged across the full sample including households at both boundaries (zero and one) of the distribution. We reported adjusted average marginal effect, representing the average change in the predicted proportion of eligible household contacts screened per 0.1 unit (10%) higher MP deprivation from its median, estimated across all households.

We selected urban/rural household location to examine effect measure modification and the following covariates for multivariable adjustment based on a priori evidence and rationale: index participant age, sex, race, birth country, household size, and HHCT strategy (Supplementary Methods 5). We compared models across covariate specifications using the Akaike Information Criterion (AIC) to confirm best fit, with the primary adjusted model consistently demonstrating the lowest AIC across all 3 outcome models (Supplementary Table 7) [25]. We estimated the intraclass correlation coefficient to determine the proportion of total variance attributable to between-clinic differences (clustering effect) [26]. As sensitivity analyses to demonstrate the robustness of our findings, we examined separate models for urban and rural households, the potential impact of alcohol consumption and smoking status, effect measure modification of HHCT strategy, and the influence of individual clinics using a leave-one-out analysis by iteratively excluding one clinic at a time and refitting our models. All analyses were performed using R v.4.3.1.

## RESULTS

The demographic characteristics of 3392 individuals diagnosed with TB in our analytic sample—including 1757 households in urban Soshanguve (52%) and 1635 households in rural Limpopo (48%)—are summarized in Table 1. Households with MP at or above the study median of 0.112 were more frequently located in rural Limpopo (75% vs 25% in urban Soshanguve), were located farther from clinics (median 25 km vs 2 km), and more frequently reported 4 or more household contact persons (41% vs 19%) and any children (40% vs 18%). Stated comfort with HHCT was high (∼97%) among all participants. Among all 3392 households, 43% initiated HHCT with at least one household contact screened, 25% had some but not all eligible contacts screened (mean proportion: 0.56 [SD: 0.16]), and 19% had all eligible contacts screened. Households with greater MP more frequently initiated HHCT (48% vs 37%), but achieved lower HHCT completion (16% vs 22%). When stratified by urban versus rural household location (Supplementary Table 8), rural households had greater poverty (ie, deprivation in an additional 17% of MP indicators), were located farther from clinics (median 25 km vs 2 km), more frequently reported 4 or more household contacts (49% vs 15%) and any children (44% vs 18%), and more frequently initiated HHCT (52% vs 35%), but achieved lower HHCT completion (14% vs 23%).

**Table 1.** Characteristics of Individuals Diagnosed With Tuberculosis in South Africa, According to Multidimensional Poverty

	Lower Poverty (MP Below Median^a^) n = 1480	Greater Poverty (MP at or Above Median^a^) n = 1912	Overall N = 3392
Age (years)^b^	43.9 (14.4)	43.6 (14.4)	43.7 (14.4)
Sex			
Female	582 (39%)	847 (44%)	1429 (42%)
Race			
Black	1477 (99%)	1898 (99%)	3375 (99%)
Birth country			
South Africa	1435 (97%)	1809 (95%)	3244 (96%)
Head of household	705 (48%)	867 (45%)	1572 (46%)
Smoking status			
Never	1092 (74%)	1366 (71%)	2458 (72%)
Former	185 (13%)	247 (13%)	432 (13%)
Current	203 (14%)	299 (16%)	502 (15%)
Alcohol consumption			
Less than 2 times per week	1437 (97%)	1777 (93%)	3214 (95%)
2–3 times per week	31 (2%)	98 (5%)	129 (4%)
4 or more times per week	12 (1%)	37 (2%)	49 (1%)
Household size^c^	3 [2, 4]	4 [3, 6]	3 [2, 5]
Number of household contacts			
1–3	1205 (81%)	1127 (59%)	2332 (69%)
4–6	232 (16%)	581 (30%)	813 (24%)
7+	43 (3%)	204 (11%)	247 (7%)
Number of children			
0	1217 (82%)	1132 (59%)	2349 (69%)
1–2	249 (17%)	695 (36%)	944 (28%)
3+	14 (1%)	85 (4%)	99 (3%)
Household distance (km) from clinic^c^	2 [1, 8]	25 [12, 40]	13 [2, 30]
Household location			
Urban Soshanguve Township	1276 (86%)	481 (25%)	1757 (52%)
Rural Limpopo Province	204 (14%)	1431 (75%)	1635 (48%)
HHCT strategy			
Standard HHCT	963 (65%)	1298 (68%)	2261 (67%)
Novel HHCT	517 (35%)	614 (32%)	1131 (33%)
Stated comfort with HHCT^d^	1438 (97%)	1840 (96%)	3278 (97%)
HHCT implementation reach^e^			
No contacts screened	931 (63%)	994 (52%)	1925 (57%)
Initiated HHCT^f^	549 (37%)	918 (48%)	1467 (43%)
Partial HHCT^g^	216 (15%)	622 (33%)	838 (25%)
Mean proportion^g^	0.58 (0.13)	0.55 (0.17)	0.56 (0.16)
Completed HHCT^h^	333 (22%)	296 (16%)	629 (19%)

Abbreviations: HHCT, household contact tracing for tuberculosis; MP, multidimensional poverty; TB, tuberculosis.

^a^Global multidimensional poverty index lower than versus equal to and greater than the median score of 0.112 (scale of 0–1, where higher scores denote greater poverty).

^b^Mean with standard deviation.

^c^Median with interquartile range.

^d^Households reporting that they were comfortable with HHCT.

^e^Reach measured as the proportion of eligible household contacts screened for TB.

^f^At least one household contact screened for TB during HHCT.

^g^Partial reach represents some but not all of eligible household contacts screened for TB. The frequency of partial reach, as well as its mean proportion and standard deviation, is reported.

^h^All eligible household contacts screened for TB during HHCT.

Table 2 presents our primary results, examining the association between MP deprivation and HHCT reach with effect measure modification by household location (urban/rural). Among all 3392 households, we found urban/rural household location to be a significant effect measure modifier (*P* = .003) of the relationship between household MP and HHCT initiation, representing at least one household contact screened. Among households in the urban township of Soshanguve, we found that each 0.1-unit higher MP (corresponding to deprivation in an additional 10% of indicators, after weighting) was associated with 10% lower prevalence of HHCT initiation (ie, adjusted prevalence ratio [aPR] 0.903; 95% confidence interval [95% CI]: 0.831, 0.984; *P* = .021). We did not observe a significant association between MP and HHCT initiation for households located in rural Limpopo Province (aPR: 1.057; 95% CI: 0.993, 1.125; *P* = .083). Among the 1467 households that initiated HHCT, we observed lower prevalence of HHCT completion (representing all household contacts screened) with greater MP (aPR: 0.945; 95% CI: 0.865, 1.032), but this association was not statistically significant (*P* = .207), and there was no effect measure modification by urban/rural household location (*P* = .113). Figure 3 illustrates these findings, with diverging slopes by urban/rural household location for HHCT initiation (representing effect measure modification) but not for HHCT completion. When we examined HHCT reach continuously across its full distribution in 3392 households, including those with no contacts screened (57%), some contacts screened (25%), and all eligible contacts screened (19%), greater MP deprivation was associated with a modest but statistically significant average reduction in the predicted proportion of eligible contacts screened (ie, adjusted average marginal effect [aAME] −1.22% [95% CI: −2.20%, −0.236%; *P* = .015] per 0.1 unit higher MP from its median). We did not observe significant effect measure modification (*P* = .072) by urban/rural household location. Figure 4 illustrates the observed modest negative slope between MP and the predicted proportion of eligible contacts screened across the full continuum of reach.

**Figure 3.**
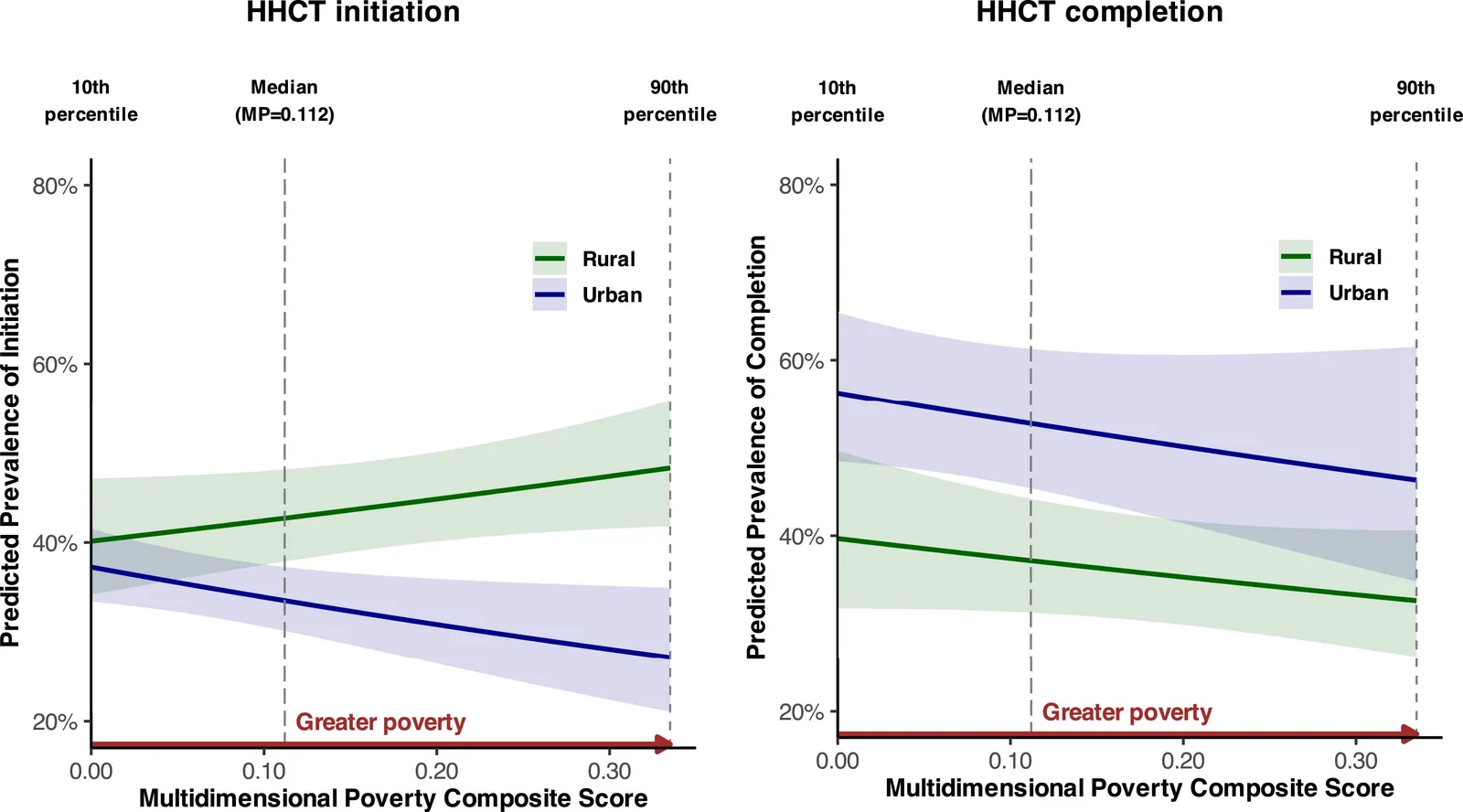
Plot of multidimensional poverty and predicted prevalence of HHCT initiation and completion. These plots present the predicted prevalence of HHCT initiation (left panel) and HHCT completion (right panel) as a function of multidimensional poverty deprivation, stratified by urban versus rural household location. The dashed vertical lines represent the 10th percentile (MP = 0), median (MP = 0.112), and 90th percentile (MP = 0.335) of the MP composite deprivation score. The left panel includes all 3392 index participants and examines whether at least one eligible household contact was screened. For HHCT initiation, greater MP deprivation was associated with significantly lower predicted prevalence among urban households (aPR = 0.903; 95% CI: 0.831, 0.984; per 0.1-unit greater MP deprivation; *P* = .021) but higher predicted prevalence among rural households (aPR = 1.06; 95% CI: 0.99, 1.13; *P* = .083). There was statistically significant effect measure modification by urban/rural household location (*P* = .003), represented by divergent slopes. The right panel is restricted to 1467 households that initiated HHCT (ie, those with at least one contact screened) and examines whether all eligible household contacts were screened. For HHCT completion, greater MP deprivation was not significantly associated with lower predicted prevalence overall (adjusted PR = 0.945; 95% CI: 0.865, 1.03; *P* = .207). There was no significant effect measure modification by urban/rural household location (*P* = .113), represented by the parallel slopes. Shading around each curve represents 95% CIs. Abbreviations: CI, confidence interval; HHCT, household contact tracing; MP, multidimensional poverty.

**Figure 4.**
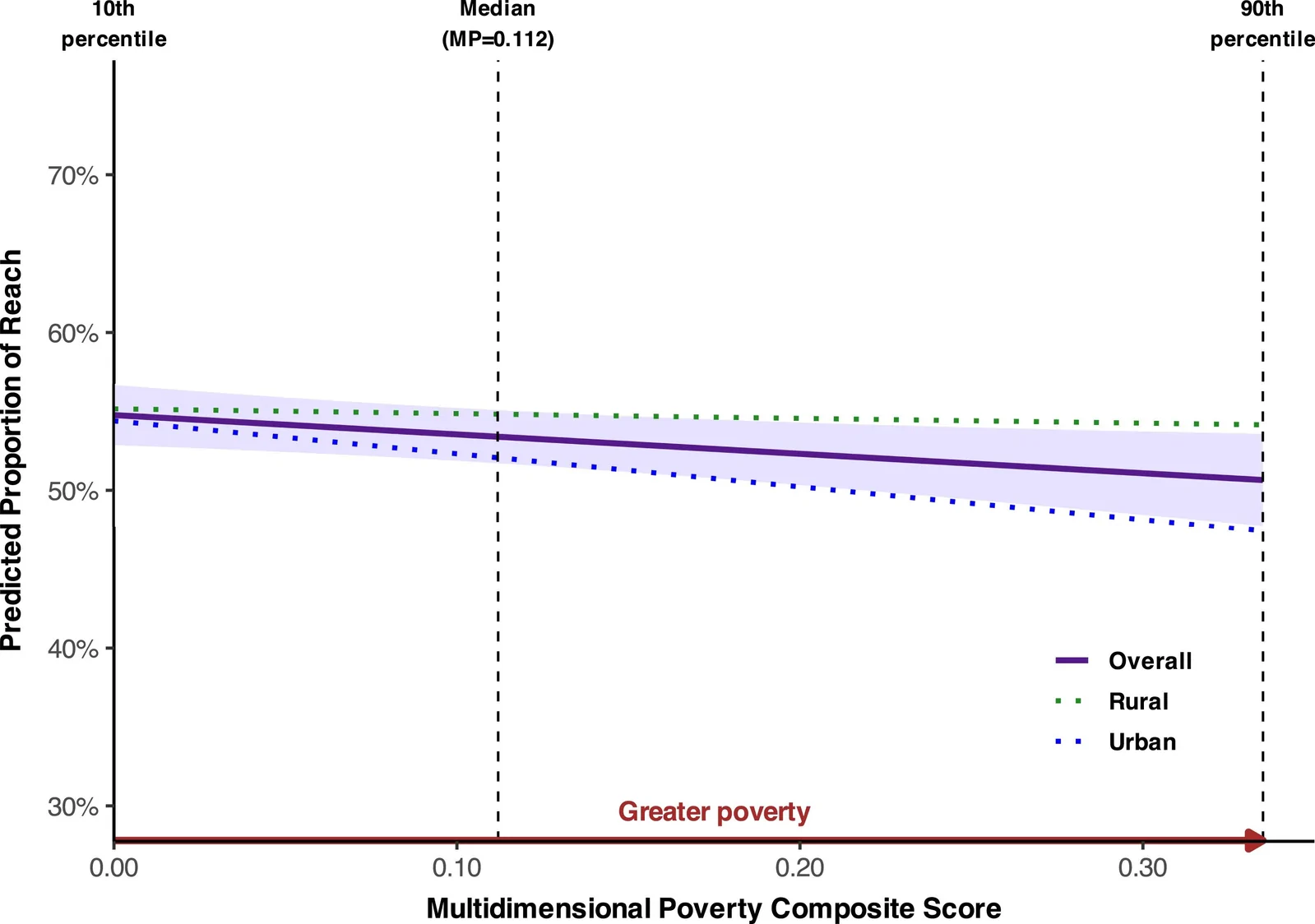
Plot of multidimensional poverty and predicted proportion of HHCT reach. This plot presents the predicted proportion of eligible household contacts screened through HHCT, a continuous outcome spanning from zero (no contacts screened) to one (all contacts screened), as a function of multidimensional poverty deprivation among all 3392 index participants. The overall marginal effect is presented in the solid line with urban/rural stratified predictions in the dotted lines. The dashed vertical lines represent the 10th percentile (MP = 0), median (MP = 0.112), and 90th percentile (MP = 0.335) of the MP composite score. Shading around the overall curve represents 95% CIs. Each 0.1-unit higher MP deprivation was associated with a modest but statistically significant 1.22% lower predicted proportion of eligible household contacts screened (95% CI: −2.20%, −0.236%; *P* = .015) with no significant effect measure modification by household location (*P* = .072). Abbreviations: CI, confidence interval; HHCT, household contact tracing; MP, multidimensional poverty.

**Table 2.** Association Between Multidimensional Poverty and Reach of Household Contact Tracing in Households With Individuals Diagnosed With Tuberculosis

	Implementation Reach of Household Contact Tracing for Each 0.1-Unit Higher Deprivation in Multidimensional Poverty	
	Adjusted Prevalence Ratio^e^	*P* Value^g^
HHCT initiation^a^		
Urban households^d^	0.903 (0.831, 0.984)	.021
Rural households^d^	1.057 (0.993, 1.125)	.083
Effect measure modification (urban vs rural households)	0.856 (0.770, 0.950)	.003
HHCT completion^b^		
All initiated households	0.945 (0.865, 1.032)	.207
Effect measure modification (urban vs rural households)	1.147 (0.969, 1.358)	.113
	Adjusted Average Marginal Effect^f^	*P* Value^g^
Average change in reach^c^		
All households	−1.22% (−2.20%, −0.236%)	.015
Effect measure modification (urban vs rural households)	−1.77% (−3.70%, 0.159%)	.072

Abbreviations: HHCT, household contact tracing for tuberculosis; MP, multidimensional poverty.

^a^HHCT initiation models were estimated among all 3392 index participants, examining whether at least one eligible household contact was screened.

^b^HHCT completion models were restricted to 1467 households that initiated HHCT (ie, at least one contact screened), examining whether all eligible household contacts were screened.

^c^Average change in proportion of reach models were estimated among all 3392 households across the full continuum of reach, including households with zero, partial, and complete screening of eligible contacts.

^d^Urban and rural stratum-specific effect estimates are presented for outcomes where statistically significant effect measure modification by urban/rural household location was detected.

^e^Adjusted prevalence ratios estimated from mixed-effects modified Poisson regression models with robust variance estimation, adjusting for index participant age, sex, race, birth country, household size, HHCT strategy with interaction by household location, and a random intercept for clinic-level clustering.

^f^Adjusted average marginal effect estimated from a mixed-effects ordered beta regression model, adjusting for index participant age, sex, race, birth country, household size, HHCT strategy with interaction by household location, and a random intercept for clinic-level clustering. This represents the average change in the predicted proportion of eligible household contacts screened per 0.1-unit higher MP deprivation averaged across all households including those with zero and complete reach.

^g^Two-sided *P* values are reported with *P* < .05 considered statistically significant. Effect measure modification *P* values reflect the interaction term between the MP composite score and urban/rural household location.

Our findings were robust across a series of sensitivity analyses. Our primary results were consistent and did not meaningfully change when we modeled urban and rural households separately (Supplementary Table 9) and adjusted for alcohol consumption and smoking status (Supplementary Table 10). There was no evidence of effect measure modification by HHCT strategy (ie, novel timing vs standard timing) for any of the MP-reach associations examined (Supplementary Table 11). Finally, we found the impact of individual clinics on observed effect estimates to be minimal (Supplementary Figure 5).

## DISCUSSION

In this analysis of data from over 3000 index participants in a randomized trial of household contact investigation for TB in northern South Africa, near-universal (97%) comfort with HHCT did not translate into high HHCT completion (19%). Greater household MP, a structural barrier, was associated with significantly lower HHCT initiation in the urban township of Soshanguve, but not in the rural districts of Vhembe and Capricorn in the Limpopo Province. Across the full continuum of reach, greater MP was associated with a modest but statistically significant reduction in the predicted proportion of eligible contacts screened. Among households that initiated HHCT, greater MP was not associated with HHCT completion.

The gap between near-universal comfort with HHCT and low HHCT completion indicates that structural and operational barriers, downstream of individual-level willingness, are likely major drivers of implementation. Multidimensional poverty, as one such structural barrier, was more strongly associated with lower HHCT initiation in the urban township site (where high levels of poverty were less prevalent) than in the rural sites (where poverty was more common). This difference may reflect different sociocultural contexts in the 2 locations. Urban townships like Soshanguve have densely populated living conditions, high transience, and heightened social pressures [27, 28]. Furthermore, in South Africa, individuals often migrate from rural to urban areas for purposes of employment [29]. The time spent engaging with healthcare workers could detract from income-generating activities, especially among casual laborers in urban settings. Living in close proximity to neighbors may also introduce social pressures when home visits are performed; these pressures may be exacerbated in poorer urban households owing to higher levels of crowding (ie, lower levels of privacy) or greater perceived differences in living conditions. In the more rural districts of Vhembe and Capricorn in Limpopo, households are more dispersed and may have more stable extended family structures, longer-term familiarity and trust with neighbors, and stronger community networks, which may provide a buffer to lessen the social pressures commonly seen in transient urban environments [30, 31].

Tuberculosis-related stigma, which presents differently based on context, may exacerbate the impact of MP on HHCT initiation. Studies surveying TB stigma across urban and rural areas in South Africa have had limited sample sizes and reported mixed findings. For example, a South African study of 397 participants reported higher TB stigma in predominantly rural Zululand, KwaZulu-Natal Province compared with urban and peri-urban areas of the Buffalo City Metropolitan district (BCM), Eastern Cape Province [32]. However, a recent study, which was one of the largest and most rigorous with 3869 households surveyed across 93 communities in BCM, found that peri-urban areas reported the highest levels of TB stigma while rural areas reported the lowest levels [33]. Similarly, within our parent trial, a small subset comprising of 143 index participants completed the perceived TB stigma survey, which appeared to find higher self-reported stigma in urban Soshanguve compared to rural Limpopo [34]. Considering data and sample limitations, we were unable to incorporate this measure of perceived TB stigma into our analysis or directly test any of these other hypotheses; future research could help to better understand the contribution of these contextual factors.

Our findings suggest that, in certain cultural contexts, successful implementation of HHCT, especially initiation, may in part depend on targeting structural barriers like MP. Integrating screening for household MP for individuals diagnosed with TB at the point of diagnosis, particularly in urban communities, may be beneficial in identifying households at risk for being missed with poor HHCT initiation. Offering targeted engagement and other interventions may help maximize HHCT initiation among multidimensionally poor urban households. Once HHCT is initiated at the household, it is likely that health system factors (ie, capacity, operations, logistics) beyond household-level MP are stronger determinants of whether all eligible contacts are screened. These factors are potentially modifiable, underscoring the need to address both household and health system-level barriers to maximize the impact of HHCT. While our analysis was rooted at the household level, future studies may focus on factors at the health system-level—including staffing, training, and resource availability—to more comprehensively inform and operationalize implementation to maximize HHCT completion.

Our findings should be interpreted in the context of certain limitations. The cross-sectional nature of our data precludes any inferences regarding the causal nature of the associations we observed. Self-reported data on poverty may also be subject to potential recall and social desirability bias. We did not measure nutritional status, one of the indicators under the health dimension in the global MPI (and an important determinant of TB risk) [35, 36], and we were therefore unable to include deprivation along this axis. Despite our inclusion of a substantial number of potential confounders, unmeasured and residual confounding remain possibilities. Finally, the HHCT intervention as designed in the trial likely excluded individuals living in communal settings (eg, jails and prisons, occupational housing, hostels, etc.) and those with transient or informal living arrangements.

## CONCLUSION

In conclusion, we found that, despite near-universal comfort with HHCT for TB, complete evaluation of all household contact persons occurred in only 1 in 5 households. Furthermore, MP—encompassing deprivations in health, education, and living standards—was associated with lower HHCT initiation in an urban South African township but not in 2 rural districts, where overall levels of poverty were higher. Across all households in both sites, greater MP was associated with a modest but significant reduction in the proportion of eligible contacts screened. These findings, taken together with those of other studies, suggest that closing the HHCT implementation gap will require addressing barriers at both the household and health system levels. Integrating screening for poverty at the point of TB diagnosis, particularly in high-density urban settings, may identify households at greatest risk of being missed and enable targeted engagement to maximize HHCT initiation. Identifying and intervening on health system factors beyond poverty may help achieve completion of HHCT once initiated. These targeted efforts are essential for successful achievement of targets to end TB (and address epidemics of other infectious and non-communicable diseases) in South Africa and similar high-burden settings.

## Supplementary Material

ciag321_Supplementary_Data

## Supplementary Data

Supplementary materials are available at *Clinical Infectious Diseases* online. Consisting of data provided by the authors to benefit the reader, the posted materials are not copyedited and are the sole responsibility of the authors, so questions or comments should be addressed to the corresponding author.
