# Supplementary material for: Multidimensional Poverty and Implementation Reach of Household Contact Tracing for Tuberculosis in South Africa: Analysis of a Randomized Trial
Source: Clin Infect Dis. 2026 Jun 17;83(3):e559–68. doi: 10.1093/cid/ciag321 (PMC13644866; doi:10.1093/cid/ciag321)
Supplement: Supplementary file 1 [file ciag321_supplementary_data.docx]

**SUPPLEMENTARY MATERIALS**

**Multidimensional Poverty and Implementation Reach of Household Contact Tracing for Tuberculosis in South Africa: Analysis of a Randomized Trial**

**Contents**

Supplementary Methods S1. Global Multidimensional Poverty Index

Supplementary Figure S1: Analytic Sample Inclusion Criteria

Supplementary Figure S2: Distribution of Outcome (HHCT Reach)

Supplementary Methods S2. Exposure Construction

Supplementary Table S1. MP Deprivation Weights

Supplementary Methods S3. Handling Missingness

Supplementary Table S2. Responses to Deprivation Indicator

Supplementary Figure S3: Pattern of Missingness

Supplementary Table S3. Participant Characteristics Missing Years of Schooling

Supplementary Table S4. Regressing Years of Schooling Missingness on Covariates

Supplementary Table S5. Regressing School Attendance Missingness on Covariates

Supplementary Table S6. Distribution of Imputed Deprivation Indicators

Supplementary Figure S4: Distribution and Centering of Exposure (MP Composite Score)

Supplementary Methods S4. Model Parameterization

Supplementary Methods S5. Variable Selection

Supplementary Table S7. Model Fitting Results

Supplementary Table S8. Participant Characteristics by Urban/Rural Household Location

Supplementary Table S9. Urban and Rural Stratified Model Results

Supplementary Table S10. Impact of Alcohol Consumption and Smoking

Supplementary Table S11. Effect Measure Modification by HHCT Strategy

Supplementary Figure S5: Leave-One-Clinic-Out Sensitivity Analysis Results

Supplementary Methods S1: Global Multidimensional Poverty

The University of Oxford’s Poverty and Human Development Initiative (OPHDI) in collaboration with the United Nations Development Programme launched the global Multidimensional Poverty Index (MPI) in 2010 to establish a more comprehensive measure of poverty beyond income. The global MPI represents an annual international measure of acute multidimensional poverty that covers over 100 developing countries.^1^ Multidimensional poverty (MP) can be defined as deprivations across three dimensions of poverty: health, education, and living standards.^1^ The global MPI encompasses acute deprivations in these three dimensions using the following 10 indicators: nutrition and child mortality (Dimension #1: Health); years of schooling and school attendance (Dimension #2: Education); cooking fuel, sanitation, drinking water, electricity, housing, assets (Dimension #3: Standards of living).^1^ OPHDI uses household questionnaire data primarily from the Demographic and Health Surveys along with Multiple Indicator Cluster Surveys to measure MPI.^1^ The Alkire-Foster method is used to construct the global MPI by first selecting dimensions and indicators informed by the Millenium Development Goals.^2,3^ Deprivation cutoffs are then applied for each indicator with relative weighting to produce a final estimate, which is compared with a cross-dimensional poverty cutoff to classify those that are multidimensionally poor.^2,3^ These estimates have been published annually based on the most recent dataset for each of the 100 countries.^2^ They undergo a rigorous harmonization process that aligns survey items and population criteria across datasets to ensure consistency over time.^2^ In addition, technical validation efforts include cross-checking deprivation indicators with DHS and MICS reports, tracking missing values to assess data completeness, and utilizing certification scripts to verify data accuracy.^2^ As a complement to the international $2.15 a day poverty rate, the global MPI is far more comprehensive in identifying how poor a household is based on their deprivation score, whether they are considered multidimensional poor based on cumulative deprivation greater than 33%, and the nature of their poverty through their deprivation profile.^1^

Supplementary Figure S1: Analytic Sample Inclusion Criteria

Supplementary Figure S2: Distribution of Outcome (HHCT Reach)

## **Supplementary Methods S2. Exposure Construction**

Our exposure was a continuous MP deprivation score based on the well-established and validated global MPI (Figure 1), with higher scores indicating greater multidimensional poverty. We identified measures of deprivation from detailed interviews of index participants and categorized them into the three dimensions of poverty with the following 10 corresponding indicators: nutrition and child mortality (Dimension #1: Health); years of schooling and school attendance (Dimension #2: Education); cooking fuel, sanitation, drinking water, electricity, housing, assets (Dimension #3: Standards of living).^1^ The original questions asked to study participants were designed using the South African MPI (SAMPI).^4^ Our analysis here constructed the broader global MPI which may be more applicable to other high-TB burden contexts. Details on construction, imputation for missingness, and visualization of distribution are found in subsequent sections.

Table S1: MP deprivation Weights

| **Global MPI Indicator** | **Study Deprivation Measure** | **Weight** | |  |
| --- | --- | --- | --- | --- |
| **Health (1/3)** | | |  |  |
| Child Mortality | At least one child within household had died | | 1/3 | |
| **Education (1/3)** | | |  |  |
| Years of Schooling | None of the household members completed 5+ years of schooling | | 1/6 |  |
| School Attendance | At least one school-age child was currently not enrolled in school | | 1/6 |  |
| **Living Standards (1/3)** | | |  |  |
| Cooking fuel | Used solid fuels for cooking | | 1/18 |  |
| Sanitation | Used chemical, pit, or bucket toilet | | 1/18 |  |
| Drinking water | Main source of drinking water was not within the dwelling or yard | | 1/18 |  |
| Electricity | No electricity for lighting and other appliance use | | 1/18 |  |
| Housing | Traditional dwelling/hut structure with dirt floor | | 1/18 |  |
| Assets | Did not own one or more: radio, TV, telephone, refrigerator, vehicle | | 1/18 |  |

For each deprivation measure we identified from our study interview questions, we assigned a deprivation score of 1 if the condition was present and 0 for no deprivation. We provided equal weight of 0.33 for each of the three dimensions, with weighting of indicators adjusted proportionally to maintain equal weighting of corresponding dimensions (Table S1). For Dimension #1 (Health), there was no available measure of malnourishment in our study, so we removed the nutrition indicator to be consistent across all households. We assigned a deprivation of 1 if the household had at least one child had died and provided 0.33 weight to this child mortality. For the education dimension, we assigned a deprivation of 1 if none of the household members completed five or more years of schooling (years of schooling) and similarly if at least one school age child was currently not enrolled in school (school attendance). For the living standards dimension, we assigned a deprivation of 1 if solid fuels was used for household cooking (cooking fuel), if the household had a chemical, pit, or bucket toilet (sanitation), if the main source of drinking water was not within the dwelling or yard (drinking water), if the household had no electricity for lighting and other appliance use (electricity), if the house was a traditional dwelling/hut structure (housing), if each household did not own one or more of the following assets: radio, TV, telephone, refrigerator, vehicle (assets).

## **Supplementary Methods S3. Handling Missingness**

We examined index participant responses to these indicators and observed some participants refused to answer, especially questions about the educational status of household members. The missingness for deprivation measures was 1% and lower for the health and living standards dimensions, but 4% and 17% for indicators in the education dimension (Table S2). In addition, we visually examined the pattern of missingness (Figure S3).

Table S2: Responses to Deprivation Indicator

| **Study Deprivation Indicators** | **Yes** | **No** | | **Refused** |
| --- | --- | --- | --- | --- |
| **Health** | | | | |
| At least one child within household had died | 68 | 3287 | 37 (1%) | |
| **Education** | | | | |
| None of the household members completed 5+ years of schooling | 617 | 2193 | 582 (17%) | |
| At least one school-age child was currently not enrolled in school | 188 | 3065 | 139 (4%) | |
| **Living Standards** | | | | |
| Used solid fuels for cooking | 1051 | 2340 | 1 (0.03%) | |
| Used chemical, pit, or bucket toilet | 2023 | 1369 | 0 | |
| Main source of drinking water was not within the dwelling or yard | 1149 | 2240 | 3 (0.09%) | |
| No electricity for lighting and other appliance use | 89 | 3292 | 11 (0.3%) | |
| Traditional dwelling/hut structure with dirt floor | 160 | 3232 | 0 | |
| Did not own one or more: radio, TV, telephone, refrigerator, vehicle | 816 | 2568 | 8 (0.2%) | |

Figure S3: Pattern of Missingness

We then examined the distribution of participants with missing years of schooling (17%) with all index participants (Table S3). Among the 528 index participants with missing data on years of schooling, we found they were mostly similar to all 3,392 index participants in our analytic sample for age, sex, race, birth country, head of household, and acceptability. Participants with missing education data were predominantly from urban households (96.9% vs. 51.8%) with shorter distance from clinic to the household (1 km vs. 13 km), and a higher proportion of 1-3 household contacts (95.5% vs. 68.8%) compared to all participants. This indicates that pattern in missingness may not be completely random to observed covariates.

Table S3: Characteristics of Participants Missing Years of Schooling Data

| **Variable** | | | **Participants missing years of schooling (n=528)** | | **All Index Participants (n=3,392)** |
| --- | --- | --- | --- | --- | --- |
| Age (mean ± SD) | | | 43.1 ± 14.8 | | 43.7 ± 14.4 |
| Sex: Female (%) | | | 39.2% | | 42.1% |
| Race: Black (%) | | | 99.7% | | 99.5% |
| Birth Country: South Africa (%) | | | 96.2% | | 95.6% |
| Head of Household: Yes (%) | | | 49.7% | | 46.3% |
| Urban Household Location (%) | | | 96.9% | | 51.8% |
| Number of Household Contacts  1-3 (%) | | | 95.5% | | 68.8% |
|  | 4-6 (%) | 4.1% | | 24% | |
|  | 7-9 (%) | 0.2% | | 5.6% | |
|  | 10+ (%) | 0.2% | | 1.7% | |
| Number of Household Children  0 (%) | | | 97.1% | | 69.3% |
| 1-2 (%) | | | 2.9% | | 27.8% |
| Household Distance from Clinic | | | 1 [1, 1] | | 13 [2, 30] |
| Acceptability (%) | | | 98.3% | | 96.6% |

We formally tested the assumption of missing completely at random (MCAR) using Little’s MCAR test, which yielded statistically significant (p<0.05) result, indicating rejection of the null hypothesis of MCAR.^5^ Therefore, we are able to confirm that the probability of missingness is not independent of both the observed and unobserved data.^6^ We proceeded to examine the assumption of missing at random (MAR), referring to missingness depending only on the observed data.^6^ We conducted a mixed-effects logistic regression analysis of the two deprivation measures with the highest level of missingness (years of schooling: 17%, school attendance: 4%), modeling the odds of missingness as a function of the other observed variables.

**Table S4: Regressing Years of Schooling Missingness on Observed Covariates**

|  | | |  | Odds Ratio (95% CI) p-value | | | | | | | |
| --- | --- | --- | --- | --- | --- | --- | --- | --- | --- | --- | --- |
| **Fixed Effects** | | |  | | |  | | | |  |  |
|  | Intercept | 0.097 (0.04, 0.22) | | | | | | |  | |  |
|  | Age (years) | 0.996 (0.99, 1.01) | | | | | | | 0.406 | |  |
|  | Female vs. Male | 1.065 (0.85, 1.34) | | | | | | | 0.589 | |  |
|  | Not Head of Household vs. Head | 1.182 (0.91, 1.54) | | | | | | | 0.215 | |  |
|  | Other Birth Country vs. South Africa | 0.547 (0.1, 2.85) | | | | | | | 0.473 | |  |
|  | Distance to Clinic (km) | 0.961 (0.95, 0.97) | | | | | | | <0.001 | |  |
|  | Number of Household Contacts | 0.595 (0.53, 0.66) | | | | | | | <0.001 | |  |
|  | Urban vs. Rural | 23.369 (11.92, 45.81) | | | | | | | <0.001 | |  |
| **Random Effects** | | | | |  | |  |  |  |  |  |
| Random Intercept Variance | | |  | | | 0.261 | | | | | |

Table S5: Regressing School Attendance Missingness on Observed Covariates

|  |  | | Odds Ratio (95% CI) | | p-value | |  | |  |
| --- | --- | --- | --- | --- | --- | --- | --- | --- | --- |
|  | **Fixed Effects** | | | | | | |  |  |
|  | Intercept | | 0.013 (0, 0.04) | |  | |  | |  |
|  | Age (years) | | 0.999 (0.98, 1.01) | | 0.875 | |  | |  |
|  | Female vs. Male | | 0.794 (0.55, 1.15) | | 0.218 | |  | |  |
|  | Not Head of Household vs. Head | | 0.773 (0.51, 1.18) | | 0.230 | |  | |  |
|  | Other Birth Country vs. South Africa | | 1.220 (0.15, 9.66) | | 0.851 | |  | |  |
|  | Distance to Clinic (km) | | 1.002 (1.001, 1.003) | | <0.001 | |  | |  |
|  | Number of Household Contacts | | 0.999 (0.9, 1.12) | | 0.991 | |  | |  |
|  | Urban vs. Rural | | 6.397 (3.64, 11.23) | | <0.001 | |  | |  |
| **Random Effects** | |  | |  | |  | | | |
|  | Random Intercept Variance | | 0.033 | |  | |  | |  |

We found the missingness of years of schooling (Table S4) and school attendance (Table S5) were both significantly associated with several observed variables (p<0.001), indicating that the mechanism of missingness in this case is highly likely to be MAR.^6^ Therefore, we selected the gold standard Multiple Imputation with Chained Equations (MICE), generating 50 imputed datasets and pooled using Rubin’s rule to obtain final imputed estimates.^7^

Table S6: Distribution of Imputed Deprivation Indicators

|  | **Original Data** | | **After Multiple Imputation** | |
| --- | --- | --- | --- | --- |
| **MPI Deprivation** | **No** | **Yes** | **No** | **Yes** |
| **Health** |  |  |  |  |
| At least one child within household had died | 3287 (98.0%) | 68 (2.0%) | 3324 (98.0%) | 68 (2.0%) |
| **Education** |  |  |  |  |
| None of the household members completed 5+ years of schooling | 2193 (78.0%) | 617 (22.0%) | 2773 (81.7%) | 619 (18.3%) |
| At least one school-age child was currently not enrolled in school | 3065 (94.2%) | 188 (5.8%) | 3199 (94.3%) | 193 (5.7%) |
| **Living Standards** |  |  |  |  |
| Used solid fuels for cooking | 2340 (69.0%) | 1051 (31.0%) | 2341 (69.0%) | 1051 (31.0%) |
| Main source of drinking water was not within the dwelling or yard | 2240 (66.1%) | 1149 (33.9%) | 2243 (66.1%) | 1149 (33.9%) |
| No electricity for lighting and other appliance use | 3292 (97.4%) | 89 (2.6%) | 3303 (97.4%) | 89 (2.6%) |
| Did not own one or more: radio, TV, telephone, refrigerator, vehicle | 2568 (75.9%) | 816 (24.1%) | 2573 (75.9%) | 819 (24.1%) |

We constructed the composite MP deprivation score by aggregating deprivation scores across the indicators for each dimension and weighted to ensure the three dimensions overall would have equal weight. We visualized the exposure and centered it at its median (MP = 0.112) owing to the right-skewness we observed and scaled by 10 for ease of interpretation.

Figure S4: Distribution and Centering of Exposure (MP score)

Supplementary Methods S4: Model Parameterization

We parameterized three mixed-effects regression models to examine the association between multidimensional poverty and HHCT implementation reach. All models included a random intercept for clinic-level clustering and were adjusted for index participant age, sex, race, birth country, household size, study arm. Effect measure modification by urban/rural household status was examined. We fit the models using maximum likelihood estimation with Laplace approximation for the random effects.

**HHCT initiation and Completion:**

We defined HHCT initiation as at least one eligible household contact screened vs. none among all 3,392 households. We defined HHCT completion as all eligible household contact screened vs. less than all among 1,467 households that have initiated HHCT. We used mixed-effects modified Poisson regression models with robust variance estimation to estimate prevalence ratios separately for initiation and completion. We included a multiplicative interaction term between MP and household location to test for effect measure modification:

$\mathrm{Log}\left( E[Yij] \right)=$ α_0_ + β_1_(MP_ij_–$\tilde{\mathrm{MP}_{\mathrm{ij}}}$) + β_2_*Urban_ij_ + β_3_(MP_ij_–$\tilde{\mathrm{MP}_{\mathrm{ij}}}$)*Urban_ij_ + β_z_X_ij_ + b_j_ + ε_ij_ for the i^th^ index participant in the j^th^ clinic (cluster), where α_0_ represents the fixed-effects intercept, MP_ij_–$\tilde{\mathrm{MP}_{\mathrm{ij}}}$ represents centering of MP at its median and subsequent scaling of the proportion by ten, β_1_ represents the fixed-effects slope (main effect of MP deprivation on reach in rural households), β_1_ + β_3_ represents the fixed-effects slope (main effect of MP deprivation on reach in urban households), β_3_ represents the interaction term, capturing effect measure modification, X_ij_ represents the vector of covariates for adjustment, b_j_ ~ N(0, σ_b_^2^) represents the random effect intercept, and ε_ij_ the residual error. Exponentiated coefficeints (e^β^) represent adjusted prevalence ratios (aPRs), representing the change in the prevalence of HHCT initiation for each 0.1 unit higher MP deprivation (from its median).

**HHCT Reach:**

We defined continuous HHCT reach as the proportion of named eligible household contacts screened in each household, bounded by and including zero and one. The observed distribution for reach exhibited inflation at both zeros (households with no contacts screened) and one (households with all eligible named contacts screened). We parametrized a mixed-effects ordered beta regression model, which accommodates the boundary inflation by jointly modeling the probability of a zero response, a one response, and a continuous proportion between zero and one using a single linear predictor and two ordered cutpoints (ζ1, ζ2). By doing so, we reduce over-parameterization of other two-part, hurdle models with higher efficiency and precision.

$g\left( \mu_{ij}] \right)=$ α_0_ + β_1_(MP_ij_–$\tilde{\mathrm{MP}_{\mathrm{ij}}}$) + β_2_*Urban_ij_ + β_3_(MP_ij_–$\tilde{\mathrm{MP}_{\mathrm{ij}}}$)*Urban_ij_ + β_z_X_ij_ + b_j_ + ε_ij_ , where g() is the logit link function applied to the conditional mean $\mu_{ij}$ of the continuous proportion among observations not at the boundaries. As the regression coefficients from ordered beta regression do not have a direct marginal interpretation on the probability scale, we estimated average marginal effects (AMEs) using numerical differentiation. The marginal effect of MP was computed as the partial derivative of the predicted proportion with respect to MP for each observation, then averaged across all households including those at the boundaries. The adjusted AME represents the average change in the predicted proportion of eligible household contacts screened for each 0.1-unit higher MP deprivation (from its median), estimated across all households.

Supplementary Methods S5: Variable Selection

We selected the following covariates for multivariable adjustment based on a priori evidence and rationale for potential association with both household MP and HHCT reach: index participant age, sex, race, birth country, household size, and HHCT strategy. We treated household location (urban/rural) as an effect measure modifier owing to contextual heterogeneity, differential needs of patient populations, and health system barriers that exist. We adjusted for index participant age, sex, race, and birth country as core demographic covariates given their established associations with both poverty and health-seeking behavior. We selected household size (i.e., index participant plus the number of household contacts) and therefore did not additionally adjust for number of household contacts and the number of children, as these are component measures. As we treated urban/rural household location as an effect measure modifier, we did not adjust for household distance from clinic owing to collinearity to preserve the heterogeneity. We adjusted for HHCT strategy, standard vs. novel HHCT, as the strategy itself may independently influence reach regardless of poverty level. We did not adjust for stated comfort with HHCT as we believe this variable to act in between the causal pathway of MP to reach. As a sensitivity analysis, we additionally examined whether the association between MP and HHCT varied by HHCT strategy. Although smoking status and alcohol consumption are plausible correlates of both poverty and health-seeking behavior, they may also act as potential mediators. Therefore, we did not include them in the primary models, which we confirmed during model testing. In sensitivity analyses, we examined their potential influence and did not find any significant impact.

Supplementary Table S7: Model Fitting Results

| **HHCT Initiation AIC** | |
| --- | --- |
| Unadjusted | 5368.30 |
| Adjusted for age, sex, race with interaction for household location | 5339.00 |
| Adjusted for Age, sex, race, birth country, household size, HHCT strategy with interaction for household location | **5287.40** |
| Age, sex, race, birth country, household size, study arm, alcohol consumption, smoking status with interaction for household location | 5294.20 |
| **HHCT Completion** | |
| Unadjusted | 2208.90 |
| Adjusted for age, sex, race with interaction for household location | 2209.70 |
| Adjusted for Age, sex, race, birth country, household size, HHCT strategy with interaction for household location | **2113.20** |
| Age, sex, race, birth country, household size, study arm, alcohol consumption, smoking status with interaction for household location | 2118.4 |
| **Average Change in Reach (%)** | |
| Unadjusted | 5892.40 |
| Age, sex, race | 5892.60 |
| Age, sex, race, birth country, household size, HHCT strategy with interaction for household location | **5838.50** |
| Age, sex, race, birth country, household size, arm, alcohol consumption, smoking status | 5843.80 |

Supplementary Table S8: Participant Characteristics by Urban/Rural Household Location

|  | **Rural Limpopo  (N=1635)** | **Urban Soshanguve (N=1757)** | **Overall (N=3392)** |
| --- | --- | --- | --- |
| **Age (years)^*^** | 43.5 (14.4) | 43.9 (14.5) | 43.7 (14.4) |
| **Male Sex** | 893 (55%) | 1070 (61%) | 1963 (58%) |
| **Black Race** | 1626 (99%) | 1749 (99%) | 3375 (99%) |
| **Birth Country** |  |  |  |
| South Africa | 1569 (96%) | 1675 (95%) | 3244 (96%) |
| Zimbabwe | 40 (2%) | 53 (3%) | 93 (3%) |
| Mozambique | 18 (1%) | 19 (1%) | 37 (1%) |
| **Head of Household** | 687 (42%) | 885 (50%) | 1572 (46%) |
| **Household Size^‡^** | 4 [3, 6] | 3 [2, 4] | 3 [2, 5] |
| **Smoking Status** |  |  |  |
| Never | 1,136 (69%) | 1,322 (75%) | 2,458 (72%) |
| Former | 235 (14%) | 197 (11%) | 432 (13%) |
| Current | 264 (16%) | 238 (14%) | 502 (15%) |
| **Alcohol Consumption** |  |  |  |
| Less than 2 times per week | 1,487 (91%) | 1,727 (98%) | 3,214 (95%) |
| 2-3 times per week | 103 (6%) | 26 (1%) | 129 (4%) |
| 4 or more times per week | 45 (3%) | 4 (0.002%) | 49 (1%) |
| **Number of Household Contacts** |  |  |  |
| 1-3 | 819 (50%) | 1513 (86%) | 2332 (69%) |
| 4-6 | 595 (36%) | 218 (12%) | 813 (24%) |
| 7-9 | 168 (10%) | 23 (1%) | 191 (6%) |
| 10+ | 53 (3%) | 3 (0.2%) | 56 (2%) |
| **Number of Children** |  |  |  |
| 0 | 917 (56%) | 1432 (82%) | 2349 (69%) |
| 1-2 | 640 (39%) | 304 (17%) | 944 (28%) |
| 3+ | 78 (5%) | 21 (1%) | 99 (3%) |
| **Household Distance from Clinic^†^** | 25 [15, 36] | 2 [1, 15] | 13 [2, 30] |
| **Multidimensional Poverty^†^** | 0.168 [0.112, 0.279] | 0 [0, 0.112] | 0.112 [0, 0.223] |
| **Stated Comfort with HHCT^§^** | 1,592 (97%) | 1,686 (96%) | 3,278 (97%) |
| **Study Arm** |  |  |  |
| Standard | 1,097 (67%) | 1,164 (66%) | 2,261 (67%) |
| Novel | 538 (33%) | 593 (34%) | 1,131 (33%) |
| **HHCT Implementation Reach^ℑ^** |  |  |  |
| No contacts screened | 783 (48%) | 1,142 (65%) | 1,925 (57%) |
| Initiated HHCT ^Ω^ | 852 (52%) | 615 (35%) | 1,467 (43%) |
| Partial HHCT^o^ | 619 (38%) | 219 (12%) | 838 (25%) |
| Mean proportion^o^ | 0.55 (0.17) | 0.58 (0.12) | 0.56 (0.16) |
| Completed HHCT**^**^** | 233 (14%) | 396 (23%) | 629 (19%) |

*Abbreviations*- MPI: Multidimensional Poverty Index; HHCT: Household Contact Tracing for Tuberculosis

**^†^**Global multidimensional poverty index lower than vs. equal to and greater than the median score of 0.112 (scale of 0 to 1, where higher scores denote greater poverty)

**^*^**Mean with standard deviation

**^‡^**Median with interquartile range

^§^Households reporting that they were comfortable with HHCT

^ℑ^Reach measured as the proportion of eligible household contacts in each household screened for TB

^Ω^At least one household contact screened for TB during HHCT

ºPartial reach represents some but not all of eligible household contacts screened for TB. The frequency of partial reach as well as its mean proportion along with standard deviation reported

**^**^**All eligible household contacts screened for TB during HHCT

Supplementary Table S9: Urban and Rural Stratified Model Results

|  | | **Implementation Reach of Household Contact Tracing**  For each 0.1-unit higher deprivation in multidimensional poverty  **Adjusted Prevalence Ratio^§^ p-value*** | | | | |  |  |
| --- | --- | --- | --- | --- | --- | --- | --- | --- |
| **HHCT Initiation** | | |  | |  | | |  |
|  | Urban stratified model | | | 0.908 (0.834, 0.989) | | 0.027 | |  |
|  | Rural stratified model | | | 1.050 (0.986, 1.119) | | 0.131 | |  |

Supplementary Table S10: Impact of Alcohol Consumption and Smoking

|  | | **Implementation Reach of Household Contact Tracing**  For each 0.1-unit higher deprivation in multidimensional poverty  **Adjusted Prevalence Ratio^§^ p-value*** | | | | | |  |  |
| --- | --- | --- | --- | --- | --- | --- | --- | --- | --- |
| **HHCT Initiation** | | |  | |  | | | |  |
|  | Urban households° | | | 0.905 (0.831, 0.985) | | 0.021 | | |  |
|  | Rural households° | | | 1.054 (0.991, 1.122) | | 0.097 | | |  |
|  | Effect measure modification (*Urban vs. rural households*) | | | 0.858 (0.772, 0.953) | | 0.004 | | |  |
|  | Current smoker vs, never | | | 1.035 (0.883, 1.215) | | 0.669 | | |  |
|  | Former smoker vs. never | | | 0.998 (0.842, 1.184) | | 0.984 | | |  |
|  | Alcohol consumption four or more times per week vs. never | | | 0.984 (0.647, 1.496) | | 0.941 | | |  |
| **HHCT Completion** | | |  | | |  | | | |
| All initiated households | | | 0.941 (0.861, 1.027) | | | 0.177 | | | |
| Effect measure modification  (*Urban vs. rural households*) | | | 1.149 (0.970, 1.361) | | | 0.109 | | | |
| Current smoker vs, never | | | 1.201 (0.959, 1.506) | | | 0.113 | | | |
| Former smoker vs. never | | | 1.137 (0.883, 1.464) | | | 0.321 | | | |
| Alcohol consumption four or  more times per week vs. never | | | 0.891 (0.419, 1.893) | | | 0.765 | | | |
|  | | | **Adjusted Average Marginal Effect** | | | | **p-value*** | | |
| **Average Change in Reach** (%) | | |  | | |  | | | |
| All households | | | -1.24% (-2.22%, -0.256%) | | | 0.013 | | | |
| Effect measure modification  (*Urban vs. rural households*) | | | -1.74% (-3.67%, 0.191%) | | | 0.077 | | | |
| Current smoker vs, never | | | 1.65% (-1.29%, 4.58%) | | | 0.705 | | | |
| Former smoker vs. never | | | 0.898% (-2.17%, 3.96%) | | | 0.965 | | | |
| Alcohol consumption four or  more times per week vs. never | | | -0.899% (-7.71%, 5.91%) | | | 0.796 | | | |

Supplementary Table S11: Effect Measure Modification by HHCT Strategy

|  | | **Implementation Reach of Household Contact Tracing**  For each 0.1-unit higher deprivation in multidimensional poverty  **Adjusted Prevalence Ratio^§^ p-value*** | | | | | | |  |  |  |
| --- | --- | --- | --- | --- | --- | --- | --- | --- | --- | --- | --- |
| **HHCT Initiation** | | |  | |  | | | | |  |  |
|  | All households  Effect measure modification (*Novel vs. standard HHCT*) | | | 1.018 (0.930, 1.115) | | 0.393 | | | |  |  |
|  | Urban households  Effect measure modification (*Novel vs. standard HHCT*) | | | 0.897 (0.731, 1.101) | | 0.301 | | | |  |  |
|  | Rural households  Effect measure modification (*Novel vs. standard HHCT*) | | | 1.040 (0.915, 1.182) | | 0.545 | | | |  |  |
| **HHCT Completion** | | |  | | |  | | | | |  |
|  | All households  Effect measure modification (*Novel vs. standard HHCT*) | | | 1.067 (0.922, 1.235) | | | 0.713 | | | | |
|  | | | **Adjusted Average Marginal Effect** | | | | | **p-value*** | | |  |
| **Average Change in Reach** (%) | | |  | | |  | | | | |  |
|  | All households  Effect measure modification (*Novel vs. standard HHCT*) | | | 0.568% (-1.03%, 2.16%) | | | 0.485 | | | | |

Supplementary Figure S5: Leave-One-Clinic-Out Sensitivity Analysis Results

**References**

1.	Global Multidimensional Poverty Index. Unpacking deprivation bundles to reduce multidimensional poverty. *United Nations Development Programme & Oxford Poverty and Human Development Initiative*. 2022;

2.	Suppa N, Kanagaratnam U. The global Multidimensional Poverty Index: Harmonised level estimates and their changes over time. *Scientific Data*. 2025/01/26 2025;12(1):153. doi:10.1038/s41597-024-04269-x

3.	Alkire S, Foster JE, Seth S, Santos ME, Roche J, Ballon P. Multidimensional poverty measurement and analysis: Chapter 5–The Alkire-Foster counting methodology. 2015;

4.	Lehohla P. The South African MPI: Creating a multidimensional poverty index using census data. 2014;

5.	Little RJ. A test of missing completely at random for multivariate data with missing values. *Journal of the American statistical Association*. 1988;83(404):1198-1202.

6.	Donders AR, van der Heijden GJ, Stijnen T, Moons KG. Review: a gentle introduction to imputation of missing values. *J Clin Epidemiol*. Oct 2006;59(10):1087-91. doi:10.1016/j.jclinepi.2006.01.014

7.	White IR, Royston P, Wood AM. Multiple imputation using chained equations: issues and guidance for practice. *Statistics in medicine*. 2011;30(4):377-399.
